# Effect of Deposition Parameters and Deposition Height on the Microstructure and Properties of Laser–Cold Metal Transfer Composite Additively Manufactured 2319 Aluminum Alloy

**DOI:** 10.3390/ma17122914

**Published:** 2024-06-14

**Authors:** Mingrui Chen, Shuncun Luo, Xiaming Chen, Xiaonan Wang, Zhikang Wu, Hiromi Nagaumi, Zengrong Hu

**Affiliations:** 1 High-Performance Metal Structural Materials Research Institute, Soochow University, Suzhou 215021, China; 2 School of Rail Transportation, Soochow University, Suzhou 215021, China; 3 School of Iron and Steel, Soochow University, Suzhou 215021, China

**Keywords:** laser arc composite additive manufacturing, deposition height, microstructure, mechanical properties

## Abstract

The 2319-Al alloy is widely used in aviation industry. The crack-free 2319 alloy thin-walled sample was fabricated utilizing the laser-CMT composite additive manufacturing technique, achieving a material utilization rate of 96.43%. The impact of deposition parameters and deposition height on the microstructure and mechanical properties was studied. The microhardness of the additive manufacturing samples exhibited a gradual decrease from construction direction, with values reaching 90 HV, 78 HV, and 72 HV, respectively. The tensile property also exhibited a gradual decrease from the bottom to the top; the highest tensile strength was 296 MPa. The grain size along the construction direction of the deposited sample gradually increased, exhibiting respective sizes of 34.7 um, 36.6 um, and 45.7 um. With the increase in the height of the second phase, the segregation at the grain boundary is intensified, and as the size inside the grain increases, the corresponding density decreases. The good laser-CMT composite additively manufactured 2319 aluminum alloy samples could be obtained under the optimized deposition parameters.

## 1. Introduction

Additive manufacturing (AM) technology, renowned for its high efficiency and low cost, is considered a promising smart manufacturing technology [1,2]. Compared to traditional processing techniques, AM technology can streamline the production process and abbreviate the processing cycle, particularly for the rapid fabrication of low-cost, complex structural components. Nowadays, AM components are primarily utilized in the aerospace, maritime, automotive, medical, and military industries [3,4].

Currently, the primary techniques employed in the field of aluminum alloy additive manufacturing technology encompass selective laser melting (SLM) and laser metal deposition (LMD), which utilize lasers as the heat source, in addition to wire + arc additive manufacturing (WAAM) [5]. SLM and LMD utilize aluminum alloy powder as the feedstock, resulting in high-precision forming and the ability to create complex structures [6]. However, there is a limited variety of aluminum alloy powders available, and removing the oxide film on the powder surface poses a challenge [7]. Additionally, the high reflectivity of aluminum alloys presents difficulties for laser processing, although this issue can be mitigated by using higher laser power. Nevertheless, excessive laser power can cause loose powder to be impacted during scanning due to the low density and lightweight nature of aluminum alloy powder, leading to the formation of pores, cracks, and other defects. The utilization of WAAM is characterized by its employment of aluminum alloy wire as raw material, which ensures deposited efficiency, as well as production costs. Additionally, it allows for the production of large-scale and complexly structured components [8]. However, the dispersion of arc energy, significant heat input, and large heat-affected zones result in low energy density, coarse microstructure, and inferior performance. In 1979, Steen from Imperial College of London initially introduced laser arc composite welding technology [9,10]. This technology combines the benefits of both heat sources, resulting in samples with superior precision, deeper melting depth, reduced heat input, and a more homogeneous structure when compared to those formed by a single arc [10,11]. The laser stabilizes and focuses the arc at the same time, which improves the accuracy of the sample. Furthermore, the arc can also reverse-stabilize the laser plasma, thereby increasing the material’s laser absorptivity. Consequently, this study combines the laser and arc heat sources to capitalize on the advantage of both additive manufacturing technologies, compensate for their respective weaknesses, and rapidly produce higher-quality alloy samples.

The Al-Cu alloy is widely used in vehicles, aerospace, and other products due to its high strength, good corrosion resistance, and excellent weldability [12,13]. At present, heat source composite welding has been extensively studied, so in this paper, a 2319 aluminum alloy was prepared by laser arc composite additive manufacturing technology. Wu et al. [14,15,16] used laser and tungsten inert gas welding (TIG) to study the additive manufacturing of aluminum alloy composites, and found that the tensile strength of 2319 aluminum alloy thin-walled parts after forming increased to 301.5 MPa compared with the strength of 221 MPa of the original material. Liu and other scholars have conducted a lot of research on laser-melt inert-gas welding (MIG) additive manufacturing of aluminum alloy, and found that the microstructure of the laser-MIG composite-formed thin-walled structure is more uniform compared with WAAM [11,17,18]. The results show that the laser arc composite additive manufacturing technology has certain advantages compared with WAAM and powder additive manufacturing. Compared with powder additive manufacturing, it has a higher deposition rate and greatly reduces the manufacturing time. At the same time, it has a high molding accuracy compared with WAAM, which ensures the utilization rate of materials and reduces the difficulty of subsequent finishing. Therefore, the process can be applied to the military field, for the very fast repair of damaged equipment, to reduce wartime costs, and to save time. At present, most studies primarily focus on the process of thin-walled aluminum alloy samples, with few investigations conducted on the relationship between the process and microstructure or the correlation between microstructure and mechanical properties [19].

In this paper, the process parameters of composite additive manufacturing and the microstructure of grain structure, second-phase morphology, and densities at different deposition heights were studied by laser and cold metal transfer (CMT) process. The microhardness and tensile properties at different heights were tested.

## 2. Materials and Experiments

**Figure 1:**
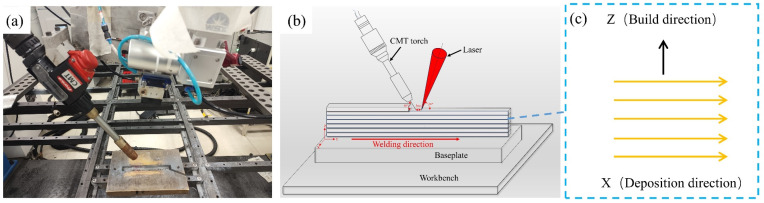
Laser-CMT composite additive manufacturing: (**a**) experimental site diagram; (**b**) schematic drawing of AM; (**c**) schematic drawing of the laser-CMT process.

The schematic diagram of laser-CMT composite additive manufacturing is presented in Figure 1. The manufacturing equipment consists of the China Beijing Keplin photoelectric Technology Co., Ltd. (Beijing, China) CWX-3000 fiber laser and a Fromius, Austria Fromius CMT TPS 500i welder (Wels, Austria). In this experiment, a laser-guided arc was employed. The angles between the welding torch and the laser, and the horizontal direction were 60° and 80°, respectively. The laser was positioned 30 cm from the substrate, with a welding wire and laser spot distance of 3 mm.

In the study, the 1.2 mm diameter 2319 aluminum alloy welding wire was selected as the raw material for additive manufacturing, with a 200 × 30 × 5 mm^3^ 2219 aluminum alloy serving as the bottom plate. The constitute of the welding wire and bottom plate is presented in Table 1. A total of 99.999% argon is used as a protective gas and velocity of 20 L/min.

Before the experiment, the bottom plate was polished with 180 sandpaper to remove the surface oxide film. Subsequent cleaning with anhydrous ethanol ensured the elimination of surface dust, grease pollutants, and other contaminants. This procedure preserved the cleanliness of the substrate surface before the addition of materials. The obtained clean substrate were used for subsequent sample accumulation to avoid the influence of surface oxides and oil stains on the experimental results.

For experimental analysis, Japan, JEOL, Ltd. (Tokyo, Japan) JSM-IT800 scanning electron microscope (SEM) was used to observe the microstructure and fracture morphology of tensile specimens. The composition of the second phase elements was determined by Japan, JEOL, Ltd. energy dispersive spectrometer (EDS). Electron back-scatter diffraction (EBSD) was used to observe the grain morphology at different heights with a scanning step of 1.5 um, and then the average grain size was calculated by the device, while the microhardness analysis was carried out using Wilson Company, Addison, TX, USA VH1102 Vickers microhardness tester with a loading force of 200 g, a holding time of 10 s, and an interval of 100 μm between each test point.

In the present investigation, the influence of laser power (*P*, W), wire feed velocity (*V_f_*, m/min), and laser scanning velocity (*V*, mm/s) on the morphology, width (*W*, mm), residual height (*H*, mm), and porosity of a single-channel molten pool were examined via single-channel and single-layer deposition tests. A total of 12 experiments were conducted with experimental methods. The experimental parameters are presented in Table 2.

**Figure 2:**
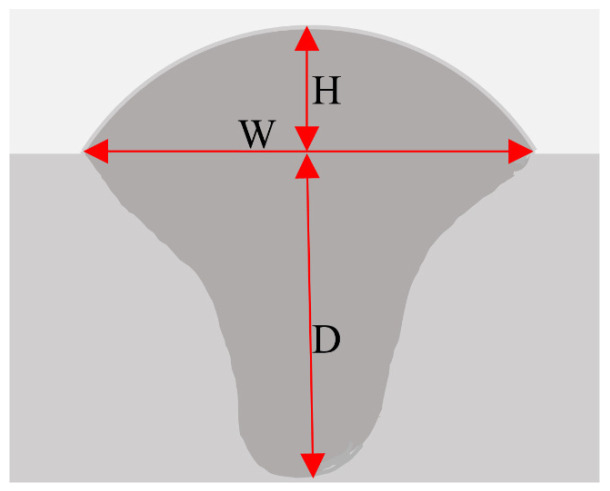
Definition of melting width (W), residual height (H), and melting depth (D).

The definitions of the weld path (W), residual height (H), and penetration depth (D) are presented in Figure 2. Based on the optimized process, parameter combination 10 (*P* = 2550 W, *V_f_* = 6 m/min, *V* = 45 mm/s) was selected for multilayer deposition. The interlayer wait time was set to 5 min to allow for deposit cooling.

**Figure 3:**
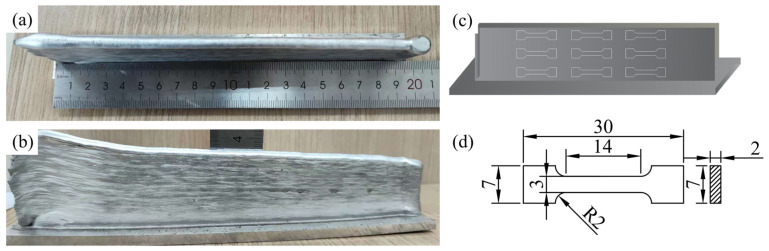
(**a**,**b**) Front view and top view of the additive manufacturing sample, (**c**) schematic diagram of the sampling position of the tensile sample, and (**d**) the size of the tensile test sample (mm).

The macroscopic additively manufacturing sample is presented in Figure 3a,b, while the sampling position diagram of the tensile sample is illustrated in (c). The diagram indicates the cut and sampling process at different deposition heights in the scanning direction. The dimensions of the tensile test sample can be observed in Figure 3d. The preparation of the tensile specimens was conducted in accordance with the Standard Room Temperature Tensile Test Method for Metal Materials (GB/T 228-2002) [20], with a total of 3 specimens at varying heights. Post tensile test, the sample’s fracture was examined.

## 3. Results and Discussion

### 3.1. Characterization of Single-Pass Morphology and Parameter Selection

**Figure 4:**
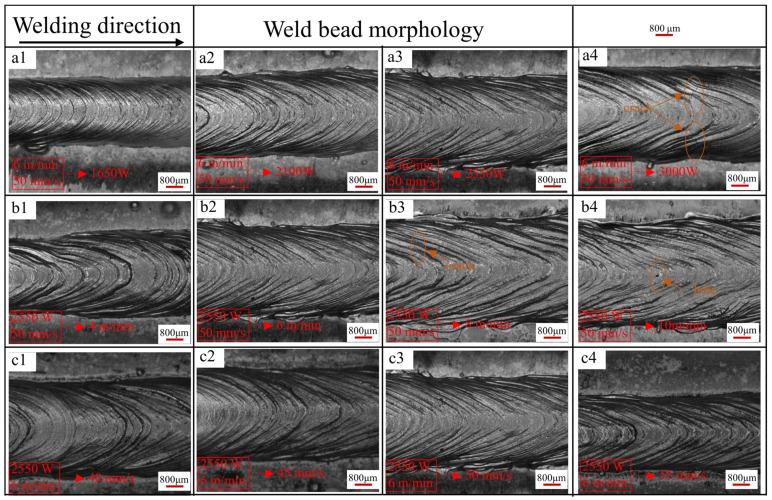
Morphologies of single-pass molten pools with different parameters: (**a1**–**a4**) wire feed speed: 6 m/min, laser scanning speed: 50 mm/s, laser power: 1650 W, 2100 W, 2550 W, 3000 W; (**b1**–**b4**) laser power: 2550 W, laser scanning speed: 50 mm/s, wire feed speed: 4 m/min, 6 m/min, 8 m/min, 10 m/min; (**c1**–**c4**) laser power: 2550 W, wire feed speed: 6m/min, laser scanning speed: 40 mm/s, 45 mm/s, 50 mm/s, 55 mm/s.

In this study, the control variable method is employed to optimize the parameters of a single molten pool [14,17,21]. As illustrated in Figure 4, the molten pool morphologies are depicted under varying powers (wire feeding speed: 6 m/min, laser scanning speed: 50 mm/s) and are presented in panels a1–a4. The solder pass morphologies under distinct wire feeding speeds (laser scanning speed: 50 mm/s, laser power: 2550 W) are presented in panels b1–b4. Finally, panels c1–c4 showcase the molten pool morphologies at varying scanning speeds (wier feeding speed: 6 m/min, laser power: 2550 W).

**Figure 5:**
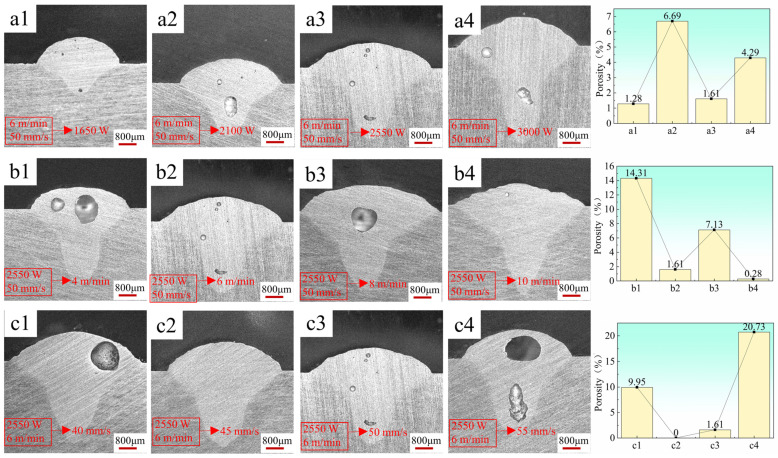
Statistics of cross-section and porosity of single molten pool with different parameters: (**a1**–**a4**) wire feed speed: 6m/min, laser scanning speed: 50 mm/s, laser power: 1650 W, 2100 W, 2550 W, 3000 W; (**b1**–**b4**) laser power: 2550 W, laser scanning speed: 50 mm/s, wire feed speed: 4 m/min, 6 m/min, 8 m/min, 10 m/min; (**c1**–**c4**) laser power: 2550 W, wire feed speed: 6 m/min, laser scanning speed: 40 mm/s, 45 mm/s, 50 mm/s, 55 mm/s.

The surface morphology of the deposition layer exhibits increasingly smooth characteristics and enhanced forming under parameter combinations a1, a2, a3, and a4 with increasing laser power. However, an increase in laser power also results in a gradual rise in heat input, leading to the emergence of cracks and pores on the sediment layer surface at a power of 3000 W, as explicated in detail in the work of J. Enz et al. [22]. At the same welding speed, the higher the laser power, the larger the length of the crack. Meanwhile, from the perspective of statistical porosity, the porosity in Figure 5(a3) is relatively low. Therefore, under the condition of a wire feed speed of 6 m/min and a laser scanning speed of 50 mm/s, the optimal laser power during formation is 2550 W.

Then, under the condition of 2550 W laser power and 50 mm/s laser scanning speed, the wire feed speed is optimized. It can be seen from Figure 4 that under parameter combination b1, the weld width is uneven due to the low wire feeding speed. With the increase in wire feeding speed, the edge burrs of the sedimentary layer gradually increase, and the flatness gradually decreases. When the wire feeding speed is 6 m/min, the overall morphology of the deposited layer is the best. When the wire feeding speed reaches 8 m/min and 10 m/min, micro-cracks and surface holes appear on the surface. Mainly due to the increase of wire feed speed, current and voltage increase, resulting in an increase in heat input [23]. At the same time, it can be noticed from the cross-section of Figure 5(b1–b4) that the porosity of parameters b2 and b4 is only 1.61% and 0.28, but surface porosity is obviously observed in Figure 4(b4) in parameter b4. At the same time, cracks have appeared on the surface when the wire feeding speed is 8 m/min, and the heat input is larger when the wire feeding speed reaches 10 m/min. Therefore, the probability of cracking is greater. Further cracks will occur in subsequent single multilayer stacking, thus making an optimal choice for wire feed rate being set at around 6 m/min.

Finally, optimizing the laser scanning speed requires controlling the laser power at 2550 W and the wire feed speed at 6 m/min. Moreover, there is insignificant variation in the surface finish of the molten pool when the laser scanning speed ranges between 40 mm/s and 50 mm/s. However, a notable decline in the forming quality of the sediment layer’s surface occurs when the wire feeding speed exceeds 50 mm/s. The escalation in laser scanning speed leads to a reduction in heat input and heat density, consequently yielding a shallow penetration depth [24]. Furthermore, the cross-section analysis of the molten pool layer reveals that at a scanning speed of 45 mm/s, porosity approaches zero. Consequently, a laser scanning speed of 45mm/s is established. The selected parameters for subsequent single multilayer stacking are as follows: laser power 2550 W; wire feed speed 6 m/min; and laser scanning speed 45 mm/s.

### 3.2. Microstructure

#### 3.2.1. Grain Structure

**Figure 6:**
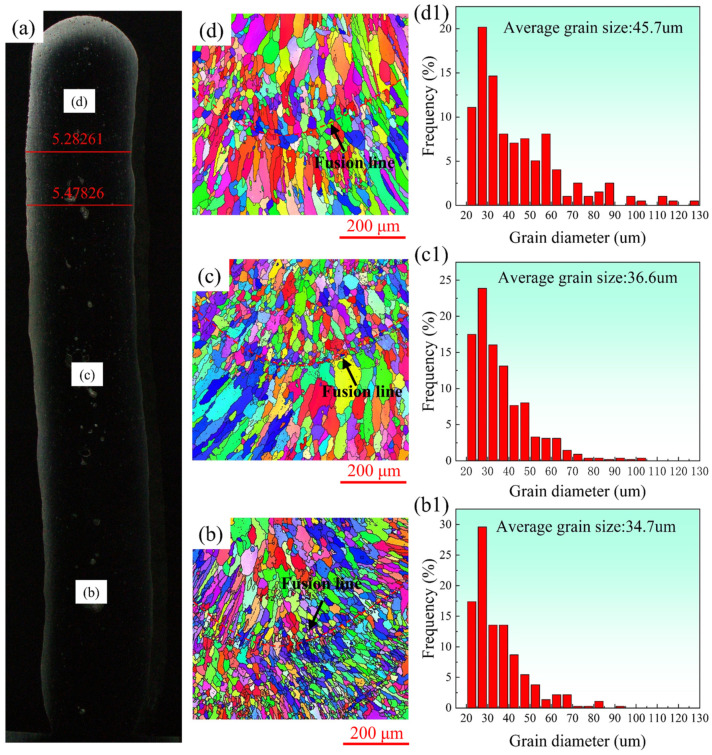
(**a**) Additive manufacturing sample cross-section; (**b**–**d**) the EBSD at the bottom, middle, and top positions, respectively; (**b1**–**d1**) grain size statistics of (**b**–**d**).

Figure 6a displays the macroscopic metallographic diagram of a thin-walled 2319 sample, fabricated via additive manufacturing techniques. The actual material utilization ratio (ηww) of thin-walled components can be expressed as follows [25]:

(1) $$\eta \mathrm{ww}=\frac{\mathrm{Eww}}{\mathrm{Mww}}$$

where Eww is the effective width, and Mww is the maximum width. Consequently, the material utilization rate of the additive-manufactured 2319 aluminum alloy sample at ηww is 96.43%. This parameter demonstrates a high level of material molding and utilization, aligning with the concept of maximizing additive manufacturing materials’ usage efficiency. Furthermore, the results suggest that the laser arc composite heat source can reduce melt pool overflow and yield good surface quality for the formed samples [26].

The microstructure of the sample, fabricated through a layer-by-layer deposition process, exhibits typical layered characteristics as depicted in Figure 6b–d. The presence of fine grains at the base of the molten pool is observed, which occurs due to the interlayer nature cooling time being meticulously controlled to 5 min during the experiment. The part that takes shape first exhibits low temperatures before the next layer is added due to the utilization of interlayer cooling [27]. Therefore, the nucleation rate is high in the remelting zone and the resultant grains are very fine [28]. However, with the increase in deposition height, the heat dissipation efficiency of the deposited layer to the substrate decreases, resulting in the accumulation of heat and the weakening of the temperature gradient between the layer and the subsequent layer, resulting in a decrease in the nucleation rate. Thus, with the increase in deposition height, the grain size in the molten pool increases and the fine crystals decrease.

It is found that the grain size in the upper region of each layer is obviously larger than that in the lower region. In addition, the gradual increase in overall grain size along the construction direction of the deposited sample is depicted in Figure 6b–d,b1–d1, exhibiting respective sizes of 34.7 um, 36.6 um, and 45.7 um. The primary cause of this phenomenon is that when gradually moving away from the base plate along the construction direction, the heat of the sample is mainly dissipated into the surrounding air, and this heat dissipation efficiency is extremely low. This leads to an accumulation of heat at higher heights and a corresponding decrease in the solidification rate of the molten pool. Consequently, there is sufficient time for grain growth to occur, resulting in an overall increase in grain size from the bottom to the top of the deposited sample.

In a research conducted by Chen et al. [29], the grain sizes of thin-walled samples fabricated from the 2319 aluminum alloy utilizing CMT + P and CMT + SP were determined to be 74 um and 61 um, respectively. In comparison with the laser-CMT additive manufacturing process employed in this study, it can be clearly seen that the grain size obtained by this technology is only 56.88% of the CMT + SP process, which highlights the obvious effect of laser stirring on grain refinement.

#### 3.2.2. The Second Phase

**Figure 7:**
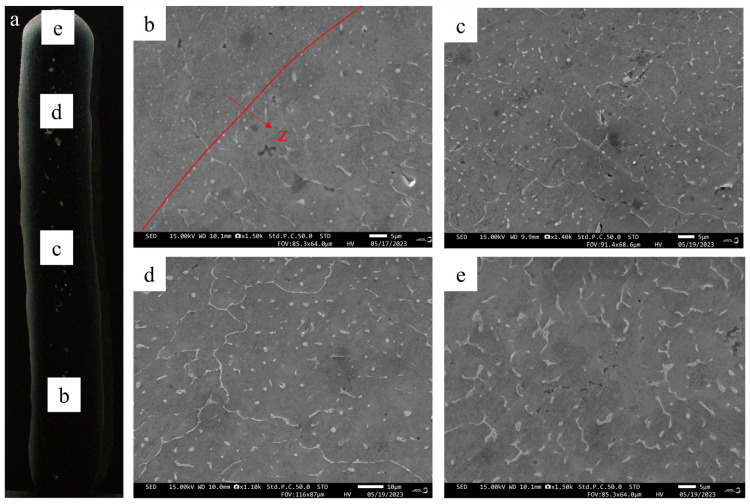
(**a**) Additive manufacturing sample cross-section; SEM images of the second phase at various locations within the additive manufacturing 2319 aluminum alloy sample: (**b**) bottom, (**c**) middle, (**d**) upper, and (**e**) the top region.

The SEM image of the deposited alloy is presented in Figure 7, depicting second-phase particles characterized by a white network. These particles are dispersed along the grain boundary or within the grain region, exhibiting distinct morphologies in various regions. As revealed in Table 3, EDS analysis indicates that the eutectic structure is composed of α-Al and Al_2_Cu (θ) phases. During the solidification process, the α-Al phase precipitates out first, with Cu-rich components being expelled from the solid–liquid interface during solidification. Upon reaching the eutectic composition ratio of Al-Cu elements at the eutectic temperature, composite eutectic particles are formed [7,30,31].

As illustrated in Figure 7b, a minor amount of θ phase is dispersed within the grain boundaries and dendrite arms at the bottom of the deposited sample, with a few spherical θ-phase particles embedded within the grains. In the central region of the deposited sample, as depicted in Figure 7c, the network of θ phase is distributed along the grain boundaries, while the spherical θ phases are dispersed within the grains. In the upper section of the deposited sample, there is an increase in the amount of reticulated θ phase and a reduction in spherical θ phase particles at the grain boundary, as illustrated in Figure 7d. As depicted in Figure 7e, at the top of the deposited sample, there is a notable coarsening of the reticulated θ phase, with only a sparse distribution of spherical θ phase particles along the grain boundaries.

The segregation in 2319 aluminum alloy during the solidification process is directly influenced by the cooling rate. As the cooling rate increases, there is a corresponding decrease in segregation [32,33]. Simultaneously, the repeated thermal cycling in the additive manufacturing process results in a decrease in cooling rate primarily due to the subsequent accumulation layers caused by an increase in height, thus reducing air heat dissipation. This results in coarsening and an uneven distribution of θ-phase particles. Furthermore, this segregation typically leads to the formation of a depletion zone around the phase by Cu-solute atoms, which can degrade the mechanical properties of the alloy [7,34].

Simultaneously, Figure 7b exhibits a notable increase in the quantity of white θ-phase particles inside the grain below the fusion line compared to above the fusion line, accompanied by smaller particle sizes compared to those observed above the fusion line [35,36]. This phenomenon is due to the heating of subsequent layers during the additive process, which involves the layered deposition of thin-walled samples followed by reheating for remelting purposes. If the cooling rate is higher at the bottom of the molten pool according to the degree of supercooling, the second phase at the bottom of the molten pool should be denser and smaller, but the opposite phenomenon occurs in Figure 7b. Therefore, due to the influence of reheating, a remelting phenomenon occurs in the previously deposited layer, during which some secondary phases dissolve and diffuse to the surrounding area. As a result, the size and quantity of θ-phase particles decreased and increased. This modification also affects the microstructure of the additive manufacturing sample [7,35,37].

**Figure 8:**
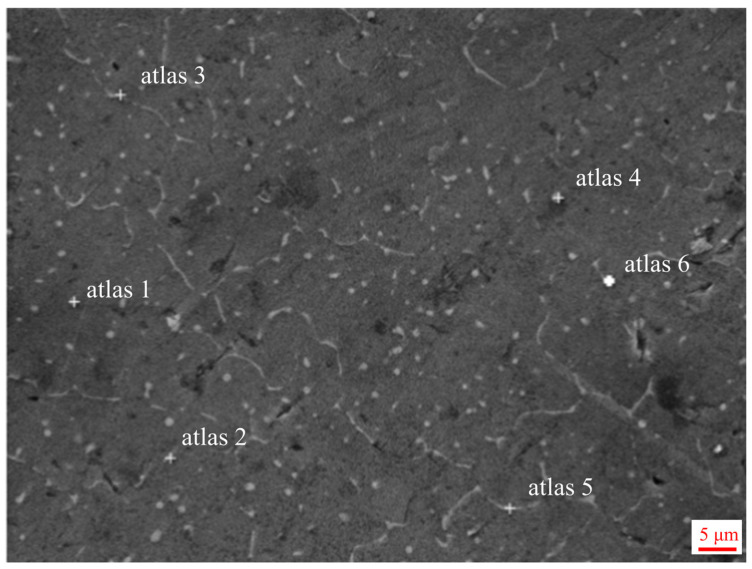
The spot scanning of the EDS: The middle region of the additive manufacturing specimen.

**Figure 9:**
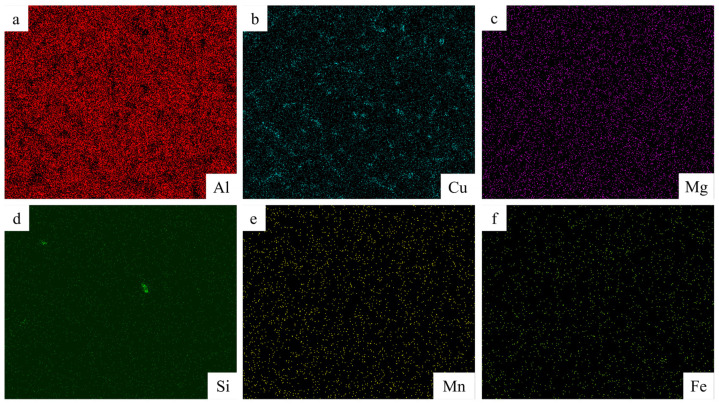
EDS scanning results of the middle region for the additive manufacturing sample: (**a**) Al, (**b**) Cu, (**c**) Mg, (**d**) Si, (**e**) Mn, (**f**) Fe.

Figure 8 shows the point scan position in the middle region of the additive sample, and the data are described in Table 3. Therefore, it is adduced from Table 3 that the second white phase is all Al_2_Cu (θ) phase. As depicted in Figure 9’s EDS scans, both Cu and Al elements are predominantly dispersed uniformly throughout the crystal structure. However, a minor portion is situated within eutectic structures at grain boundaries with discontinuous distribution patterns. Furthermore, no evidence of element burning or enrichment/deficiency regions is observed.

### 3.3. Mechanical Properties

#### 3.3.1. Microhardness

**Figure 10:**
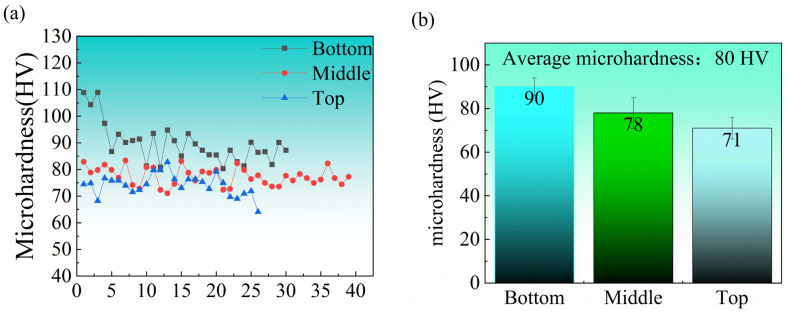
Microhardness of different positions in the additive manufacturing sample: (**a**) point plot; (**b**) bar chart.

The distribution of sample microhardness along the construction direction is shown in Figure 10. The average microhardness of the samples is 81 HV. Specifically, the average hardness at the bottom is 90 HV, while it decreases to 78 HV in the middle and further drops to 72 HV at the top.

The variation in hardness of deposited samples at different heights can be attributed to the gradual coarsening of grains with increasing deposition height, as indicated by EBSD grain size statistics in Figure 6. Furthermore, the segregation of θ particles along grain boundaries and the coarsening of circular θ particles within grains contribute to an uneven distribution of these phases. It is also the cause of changes in microhardness [37,38]. Consequently, it is evident that deposition height plays a significant role in influencing microhardness.

#### 3.3.2. Tensile Properties and Fracture Morphology

**Figure 11:**
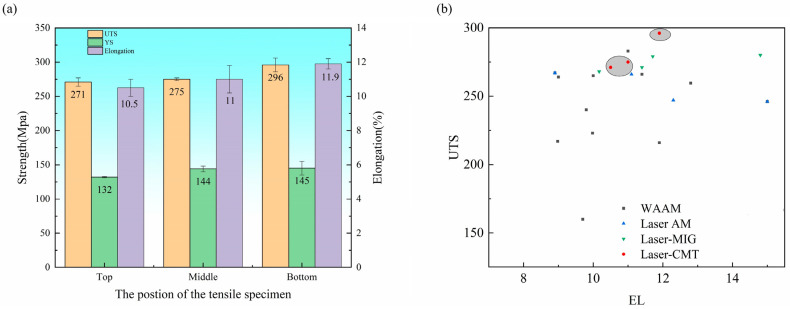
(**a**) Tensile properties of different tensile parts of thin-walled parts made by additive manufacturing; (**b**) comparison of tensile properties (The grey part is the data studied in this paper) [13,19,29,34,36,39,40,41].

The tensile properties of different parts of the deposited samples are shown in Figure 11. The bottom section of the additive thin-walled parts demonstrates an ultimate tensile strength (UTS) of 296 MPa and a yield strength (YS) of 145 MPa, accompanied by an elongation (EL) rate of 11.9%. In comparison, the middle region exhibits values of 275 MPa (UTS), 144 MPa (YS), and 11% (EL). The top part presents a UTS and YS of 271 MPa and 132 MPa, respectively, along with an EL of 10.5%.

As mentioned previously, the grain size gradually increases with the deposition height of the sample. Smaller grain sizes contribute to higher strength in metal materials due to an increased grain boundary area between these smaller grains, which effectively impedes dislocation slip and enhances tensile strength. Also, a larger grain boundary area facilitates more dislocation slip paths, thereby improving material plasticity. Moreover, the cooling rate decreases with an increase in the deposition sample height, resulting in enhanced segregation of Cu elements at the grain boundary and the coarsening and uneven distribution of θ-phase particles that renders the material susceptible to fracture. The grain boundary segregation can induce stress concentration within the grain, ultimately diminishing both strength and toughness. Consequently, the mechanical properties of the deposited samples gradually decline with increasing deposition height.

**Figure 12:**
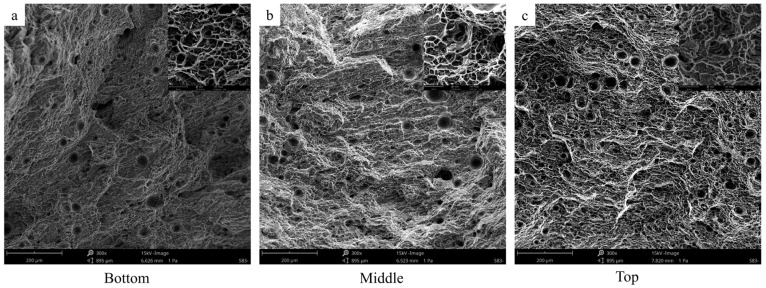
SEM morphology of tensile samples in thin-walled parts with different deposition heights: (**a**) at the bottom; (**b**) in the middle; (**c**) at the top.

Fracture morphology analysis using Figure 12 reveals numerous pores at various positions along the fracture surfaces of additive thin-walled parts, which may reduce their strength and plasticity [30]. These pore formations resemble the hydrogen pores discovered by Zhu et al. [42]. The primary cause of such porosity is attributed to the presence of hydrogen, which originates from the welding wire or surrounding air sources due to its higher solubility levels in pure liquid aluminum compared to solid aluminum [43]. Additionally, as observed in Figure 12, a significant number of dimples are present horizontally across the fracture surfaces, indicating the ductile fracture behavior exhibited by the test samples.

## 4. Conclusions

To investigate the influence of additive manufacturing parameters on weld bead formation, in this study, CMT and laser were employed as heat sources, utilizing a 2319 aluminum alloy welding wire as the raw material for single-pass deposition. Moreover, a single multilayer deposition was performed to examine the effect of deposition height on the microstructure and mechanical properties of the additive manufacturing samples. The important results of this study are as follows:

(1) The crack-free 2319 alloy thin-walled sample was fabricated utilizing the laser-CMT composite additive manufacturing technique, achieving a material utilization rate of 96.43%. This high material utilization rate aligns with the goal of maximizing additive manufacturing materials, enhancing its overall efficiency and sustainability.
(2) Along construction direction, the segregation of the secondary phase at the grain boundary intensified and its size gradually increased, while its particle density within the grain decreased. Additionally, in the direction of construction, the grain size increases gradually. The Cu element exhibited enrichment in the additive manufacturing sample.
(3) The microhardness of the additive manufacturing samples exhibited a gradual decrease from construction direction, with values reaching 90 HV, 78 HV, and 72 HV, respectively. The mechanical properties also decrease with increasing height. The tensile property also demonstrates a gradual decrease from the bottom to the top, with the highest attainable tensile strength reaching 296 MPa. At the same time, its tensile strength is higher than that of other processes; therefore, the process is feasible. And the 2319 aluminum alloy sheet is successfully prepared using laser-CMT composite additive manufacturing ductile fracture.

## Figures and Tables

**Table 1:** Constitute of welding wire and bottom plate (wt%) and material strength (MPa).

Materials	Si	Mg	Fe	Mn	Cr	Cu	Zn	Ti	Al	б_Max_
ER2319	0.2	0.2–0.4	≤0.30	0.2–0.4	-	5.8–6.8	≤0.10	0.1–0.2	Bal	221
AA2219	0.2	0.2–0.4	0.3	0.2–0.4	-	5.8–6.8	≤0.10	0.02–0.1		400

**Table 2:** Combination of test parameters for single-channel and single-layer deposition.

No.	*P* (W)	*V_f_* (m/min)	*V* (mm/s)
a1	1650		
a2	2100	6	50
a3	2550		
a4	3000		
b1		4	
b2	2550	6	50
b3		8	
b4		10	
c1			40
c2	2550	6	45
c3			50
c4			55

**Table 3:** The EDS point scanning results of Figure 8.

	Atlas 1	Atlas 2	Atlas 3	Atlas 4	Atlas 5	Atlas 6
Al	75.01	74.23	90.16	87.35	89.46	91.29
Cu	24.36	21.87	9.44	12.06	10.14	8.02
Mg	0.01	0.00	0.00	0.00	0.00	0.00
Si	0.38	0.58	0.18	0.23	0.19	0.14
Fe	0.05	2.15	0.07	0.03	0.00	0.21

## Data Availability

Data are contained within the article.
